# Optimisation of soil washing method for removal of petroleum hydrocarbons from contaminated soil around oil storage tanks using response surface methodology

**DOI:** 10.1038/s41598-023-42777-9

**Published:** 2023-09-19

**Authors:** Pouyan Zoghi, Roya Mafigholami

**Affiliations:** Department of Environment, West Tehran Branch, Islamic Azad University, Tehran, Iran

**Keywords:** Environmental sciences, Chemistry

## Abstract

Total petroleum hydrocarbons (TPHs), which are often found in soil, water, sediments, and air. These compounds are a type of pollutant that can have a serious negative impact on living things and human health. Soil washing method is a remediation technique used to remove contaminants from the soil. This process involves the use of water or other solvents to extract contaminants from the soil, followed by separation and disposal of the contaminated solution. This research engineered the effectiveness of soil washing method to remove TPHs from a genuine, sullied soil sample. After analyzing the physical and chemical properties of the soil, the Box-Benken Design (BBD) technique was used to optimize the variables that influence the process's effectiveness. A quadratic model was suggested based on the BBD design, correlation coefficients, and other factors. The minimum, maximum and mean removal of TPHs during the stages of the study were 63.5, 94.5 and 76.7%, respectively. The correlation between the variables was strong, as shown by the analysis of variance (ANOVA), F-value (1064.5) and P-value (0.0001), and the proposed model was highly significant. The most effective soil washing method (SWM) was obtained with pH 7.8, liquid to solid ratio 50:1, reaction time 52 min, surfactant concentration 7.9 mg kg^−1^, and three washings. A removal rate of 98.8% was accomplished for TPHs from the soil in this context. The kinetic results indicate that the kinetic of TPHs removal follows the first-order kinetics (R^2^ = 0.96). There was not a major difference in the process's efficiency based on temperature. The removal efficiency heightened from 0 to 150 rpm and then remained steady. Introducing air flow increased the rate of removal, and the combination of ultrasonic waves with the reaction environment increased the process efficiency and decreased the time for the process and the amount of times it needed to be washed. An analysis of the washed soil both physically and chemically revealed a substantial decrease in the concentration of other elements.

## Introduction

The rapid development of industrialization and urbanization has created soil contaminated with organic pollutants and heavy metals^1^. A wide range of goods and consumer products are manufactured by TPHs in many industries. In 2030, TPHs consumption will increase to 106.6 million barrels from 85 million barrels in 2016^2^. Petroleum components are commonly released into the environment during petroleum exploration, accidents, transportation, and leaks from waste disposal or storage sites^3^. Near Tehran oil refinery, nearly 1,500,000 m^3^ of soil were contaminated by crude oil, according to a report^4^. Among the organic pollutants found in petroleum are alkanes, cycloalkanes, polycyclic aromatic hydrocarbons, and many other cyclic hydrocarbons. The stability and durability of these pollutants make them major environmental pollutants^5^. Many pollutants found in TPHs are harmful to the environment and difficult to degrade. Over time, these contaminants exceed their background levels and gradually migrate throughout ecosystems, causing severe environmental problems, ecological disasters, and social catastrophes worldwide. Petroleum hydrocarbon-contaminated soils have been remedied using a variety of physical, chemical, and biological methods. One of the most efficient methods of removing pollutants from soil is the SWM, which involves desorption, chelation, dissolution, and other chemical processes with the addition of specific solutions. Its advantages include flexible application, simple operation, short duration, low cost, and high removal efficiency^6^. An overpressure hydraulic head pushes chemicals into contaminated soil, followed by separation and treatment to remove the liquid. Soil washing is affected by numerous factors, including environmental conditions, contaminants and extraction solutions' bonding and chelating strength. As a result, it is very important to select the right cleaning agents^7^. TPHs in an eluent are always mobilized by additives, even when the soil has low water solubility. Inorganic acids, bases, salts, and surfactants are common additives. Different additives have significantly different removal efficiencies for TPHs. By adding residual washing agents, soil washing technology increases environmental risks, changes the soil's physical–chemical properties, and destroys the ecological structure. For washing technology, therefore, it is crucial to select a washing agent that removes TPHs efficiently, is non-toxic, and does not damage soil properties^8,9^. In the soil–water system, surfactants enhance the solubility of organic compounds in aqueous solutions. There are three categories of surfactants: anionic, cationic, and nonionic. Each of them releases its electric charge when it comes in contact with water. Among the main factors that determine surfactant performance and effectiveness are its chemical nature and its selective concentration, which is determined by its critical micelle concentration (CMC)^10^. Nonionic and/or anionic surfactants are generally chosen. The biodegradability and toxicity of surfactants are also crucial parameters. Sodium dodecyl sulfate (SDS), is an organic compound with the formula CH_3_(CH_2_)_11_OSO_3_Na. There are many cleaning and hygiene products that contain anionic surfactants. It consists of sodium salts of 12-carbon organosulfates. As a detergent, this compound is amphiphilic due to its hydrocarbon tail and polar head group^11^. SDS were investigated for their effectiveness as contaminant removers, solvent recyclers, and cost-effective surfactant sources. Multivariate and their interactions are evaluated through fewer experimental trials due to response surface methodology (RSM). Among the principal RSM used in environment researches, BBD is the standard design^12^. A sandy soil contaminated with 9000 mg L^−1^ of cresols achieved 78.6% efficiency with an SDS solution according to Gitipour, and Khalladi found that a SDS solution at a concentration of 576 mg L^−1^ could remove diesel at a rate of 97% in soil with 94% silt^13,14^. The objective of this study is to improve the soil washing process to remediation of soil contaminated with TPHs based on the explanations provided. BBD applied to design the experiment, analyze the data, and determine the optimal conditions for influencing variables on soil washing (such as washing solution pH, liquid to soil ratio, SDS concentration, retention time and multi-step washing). In the optimum condition, the effect of stirring speed, airflow rate, temperature and sonication were investigated.

## Materials and methods

### Soil sampling, preparation and characterization

This investigation used soil samples sourced from near oil storage tanks in Tehran. In order to collect soil samples, standard methods based on authoritative references were used^15^. A geological information, and other features of the site can be seen in (Table S1). The soil samples were mixed and air-dried for 3–4 days at room temperature after collection, and homogenization was done in the laboratory through the use of a soil mixer. Samples of soil were sifted through a 2 mm sieve before examination. The soil sample was examined for physico-chemical properties and other desirable characteristics (Table 1). To ensure a significant relationship between the soil pollution near the storage tanks and the concentration of TPHs, control samples were taken from the distances of 500 and 1000 m from the storage tanks. The low concentration of the samples at distances of 500 and 1000 m indicated the relationship between the storage reservoirs and the concentration of TPHs.

**Table 1:** Soil physico-chemical properties and other characteristics.

Parameters	pH	Carbon	EC	Soil texture	Clay	Silt	Sand	Soil texture	TN	Potassium
Unit	–	%	DS/m	–	%					Ppm
Value	7.21	2.34	1.698	Sand-loam	6	14	80	Sand-loam	0.23	500.6

Parameters	Phosphorus	Mg	TPHs	Fe	Cd	Mn	Pb	Zn	As	
Unit	Ppm									
Value	570	46.19	250	1961	ND	300.45	275	395.1	127	

### Reagents and equipment

Sodium dodecyl sulfate (SDS, CH_3_(CH_2_)_11_OSO_3_Na, 99% purity), sulfuric acid (H_2_SO_4_, 98% purity), sodium hydroxide (NaOH, 98% purity), dichloromethane (CH_2_Cl_2_, > 99.0%), methanol (CH_3_OH, > 99.0%), and sodium sulfate (Na_2_SO_4_, > 98.5%) were purchased from Merck, Samchun, and Sigma-Aldrich Co. and used with no purification. A pH meter (Portable pH meter PH-870), a digital laboratory scale (Digital Lab Science Electronic Balance, 220 g × 0.0001 g), a mechanical mixer (VONOYA Electric Overhead Stirrer, Digital Auto Stirrer with Time Setting & Speed Adjusting, 0–3000 RPM), pipet (Cole-Parmer Essentials Adjustable-Volume Pipettor, 100 to 1000 µL), centrifuge (AP9756 Economy), syringe filter (Whatman®, USA), laboratory glassware, ultrasonic Processor (60 kHz, 350 W, Qsonica 408), aeration pumps (Pondmaster AP-8 Air Pump 545 CFM), and a gas chromatograph (Agilent Technologies, model 6890 N Network GC system, USA)-flame ionization detector (FID) were used.

### Installing SWM reactor

**Figure 1:**
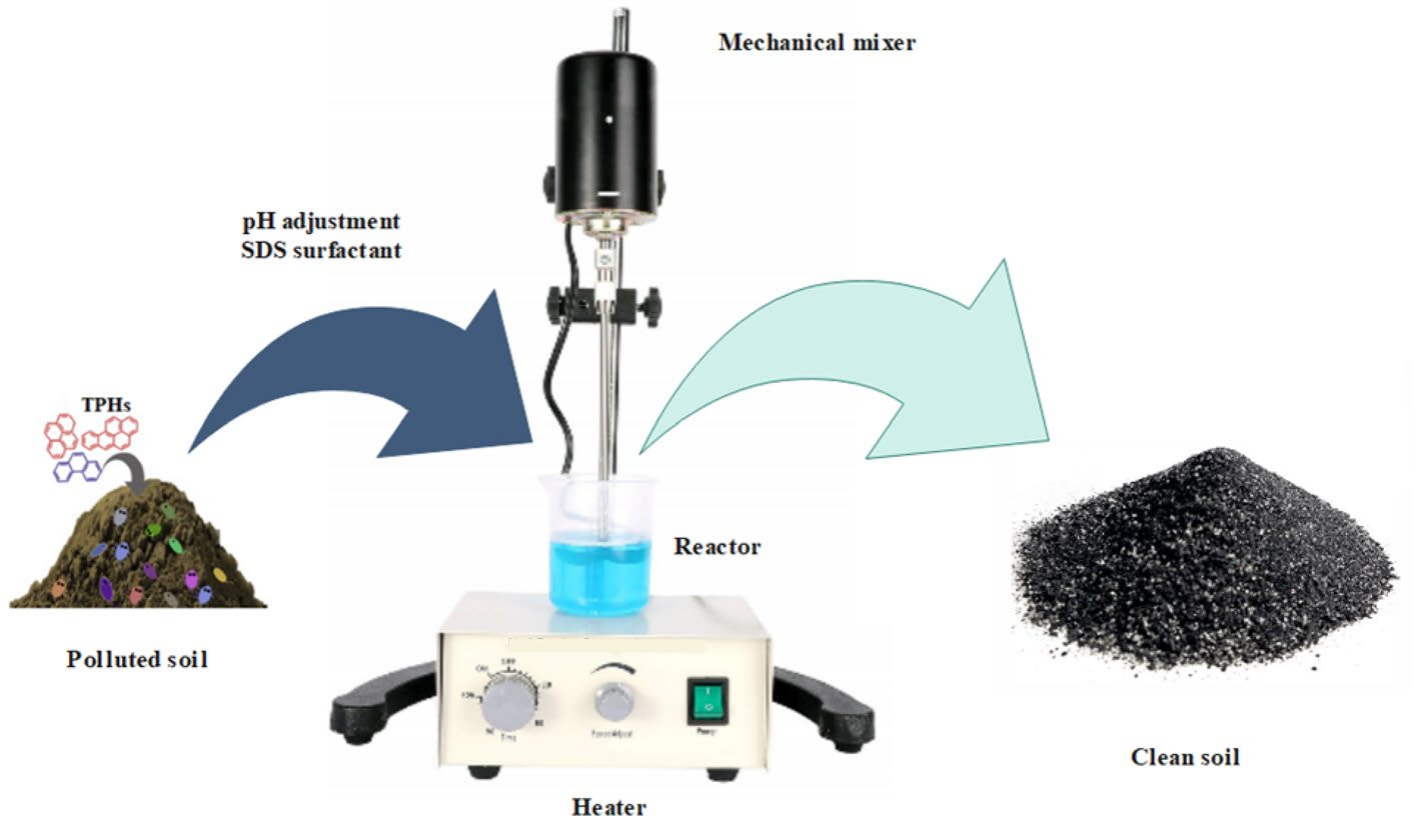
Diagram of the SWM.

A 500 mL pelxi glass cylindrical unit with a diameter of 10 cm and a height of 20 cm was utilized for the SWM runs (Fig. 1). The runs were administered using a batch system at an ambient temperature of 25 ± 2 °C. One g of contaminated soil was transferred into the reactor with 400 mL deionized-water. The reactor was equipped with the mechanical mixer, that continually mixed with 100 rpm. An air pump was used to pass air through, introducing a stable flow of air into the reaction medium by a micro-diffuser. Acid sulfuric and sodium hydroxide used to change the unique value of pH. For the measurement of TPHs, after preparing method (2–5 section), injected into GC-FID. The mean values were obtained by averaging the results of the three trials of each test. The TPHs reduction rates in the SWM were calculated with the following equation [Eq. (1)]^16^.

1 $$ {\text{Efficency}}\;{\text{removal}}\;{\text{(\% )}} = \left[ {\frac{{{\text{C}}_{{0}} - C_{t} }}{{C_{0} }}} \right] \times 100 $$

where, C_o_ and C_t_ are concentration of TPHs (ppm) in the different reaction time, respectively.

### Optimization of SWM variables based on BBD

To optimize the SWM for the removal of TPHs from polluted soil, through the BBD approach in the response surface method. Table 2 outlines the range of variables.

**Table 2:** The impact of various factors on SWM.

Factor	Name	Units	Minimum	Maximum	Coded low	Coded high	Mean	Std. dev
A	Washing solution pH	–	4	8	− 1 ↔ 4	+ 1 ↔ 8	6	1.19
B	Liquid/ Soil Ratio	–	20:1	60:1	− 1 ↔ 20:1	+ 1 ↔ 60:1	40:1	11.93
C	Surfactant concentration	mg/kg	5	10	− 1 ↔ 5	+ 1 ↔ 10	7.5	1.49
D	Washing cycles	–	1	3	− 1 ↔ 1	+ 1 ↔ 3	2	0.5963
E	Retention time	min	30	60	− 1 ↔ 30	+ 1 ↔ 60	45	8.94

The BBD design was the basis for the 46 runs that are represented in Table S.2, which were to remove TPHs.

The removal of TPHs from soil via SWM is represented by using a second-order polynomial model [Eq. (2)]^17^:

2 $$ Y = \beta_{0} + \sum\limits_{i = 1}^{k} {\beta_{i} } x_{i} + \sum\limits_{i = 1}^{k} {\beta_{ii} } x_{i}^{2} + \sum\limits_{i = 1}^{k - 1} {\sum\limits_{j = i + 1}^{k} {\beta_{ij} } } x_{i} x_{j} + \varepsilon $$

Here, Y is TPHs removal (%), β_0_ is the intercept, β_i_, β_ii_, and β_ij_ are the linear, quadratic, and interaction effect coefficients of variables respectively, x_i_ and x_j_ are coded testing classes of the variables, k is the number of the independent variables, and e is the residual error. An individual numerical value was computed for each variable with [Eq. (3)] to allow for comparison between factors with diverse units.

3 $$ x_{i} = \frac{{X_{i} - X_{0} }}{\Delta x} $$

The coded value of the variable is X_i_, the gap between the highest and lowest values of the variable is ∆_x_, and X_0_ personifies the lowest value of the variable. ANOVA was conducted by applying the P-values and F-values to assess the interaction between response and factors. R^2^ and R^2^_adj_ are correlation coefficients. In addition, R^2^ predicts were used.

### Analytical methods

TPHs levels in the soils were measured in accordance with the standard test method^18^. Initially, anhydrous Na_2_SO_4_ was blended with the gained soil to impede the infusion of water into the capillary column (Agilent, 30 m × 0.25 mm HP-5, USA). Subsequently, the soil samples were blended with 100 mL of a compound consisting of dichloromethane (CH_2_Cl_2_, > 99.0%) and methanol (CH_3_OH, > 99.0%) (3:1 volume ratio). The ultrasonic extractor was used three times for three minutes to extract the desired material, after which it was filtered through a 0.2-μm polytetrafluoroethylene (PTFE) syringe filter to separate the solid particles. Following this, a GC-FID was utilized to measure the quantity of TPHs in the solution. The column was operated with nitrogen of the highest purity as carrier gas with an initial flow rate of 2.0 mL min^−1^ and a post-run flow rate of 0.7 mL min^−1^. The oven maintained a temperature of 45 °C for 2 min before rising to 300 °C with a progression of 8 °C min^–1^.

### Supplementary investigation

#### Reaction kinetics

The TPHs removal from the soil in the optimal condition of the SWM was showed by first- and second-order model used were expressed by the following equations, respectively^14^.

4 $$ {\text{C}}_{{\text{t}}} = {\text{C}}_{{\text{i}}} {\text{exp}}\left( { - {\text{K}}_{{1}} {\text{t}}} \right) $$

5 $$ {1}/{\text{C}}_{{\text{t}}} = {\text{2K}}_{{2}} {\text{t}} + {1}/{\text{C}}_{{\text{i}}} $$

where C_t_ (ppm), is the TPHs concentration in soil at instant t, C_i_ (ppm) is the initial concentration of TPHs, k_1_ and k_2_ are the rate constants of the first and second order expressed in (min) and (ppm g^−1^ min^−1^), respectively, t (min) is the time of washing.

#### Effect of temperature, mixing speed, air flow and sonication on SWM performance

The effect of reactor temperature (20–40 °C), mixing speed (50–300 rpm), airflow entering the reactor (0.1–1 L min^−1^), and sonication (60 kHz with power 350 W) on the efficiency of the SWM when operating in the optimal conditions, was investigated.

#### Effect of soil washing on physico-chemical soil properties

After washing the polluted soil, the physical and chemical characteristics of the soil were re-examined and compared with the initial values.

## Results

### Development of a BBD, and statistical analysis

The TPHs removal results were assessed statistically to create a response surface model and identify the best conditions for SWM (Table S2). Based on the results, the minimum, maximum and mean TPHs removal in SWM values were 63.5, 94.5 and 76.7%, respectively. According to fitting the model, quadratic model suggested (Table 3).

**Table 3:** A summarized view of the model's performance.

Source	Sequential p-value	Lack of Fit p-value	Adjusted R^2^	Predicted R^2^	
Linear	< 0.0001	0/0225	0/9687	0/9646	
2FI	0/9992	0/0120	0/9600	0/9411	
**Quadratic**	** < 0.0001**	**0/9906**	**0/9979**	**0/9968**	**Suggested**
Cubic	0/9864	0/8451	0/9963	0/9847	Aliased

Significant values are in bold.

By using the ANOVA, the results of TPHs (Table S3) showed to be highly dependable and had a very low probability value for the quadratic regression model, demonstrating that it could accurately explain the codes within the actual data and predicted values. This model was incredibly valid and proficient at predicting responses, as indicated by the correlation coefficients (R^2^, R^2^_adj_, and R^2^ predict). High correlation is present between R^2^, R^2^_adj_, and R^2^ predict, indicating that the model can predict responses^19,20^. The F-value and p-value in the ANOVA analysis are reliable statistical indicators for determining the data deviation factors. These indexes led to the selection of a substantial statistical model with a high F-value and a low p-value (≤ 0.05). Fisher's F-test showed that a quadratic model would be the most suitable to fit the relationship between the predicted and experimental values of TPHs removal. The F-value of TPHs has been determined to be 1064.5 and the p-values (0.0001) provide clear evidence of an effective fit between the experimental and the expected response values. An Adequate Precision ratio (117.1) indicates an acceptable model^21^. Based on the ANOVA results, it was found that due to the physical nature of SWM, there is no significant relationship between the variables, so the mathematical relationship is simplified. The equation for the percentage of TPHs removal in SWM was expressed in terms of coded factors:

6 $$ {\text{TPHs removal }}\left( \% \right) \, = {74}/{75 } + { 5}/0{4} \times {\text{A }} + { 5}/0{2} \times {\text{B }} + { 7}/{4} \times {\text{C }} + { 4}/{9} \times {\text{D }} + { 7}/{7} \times {\text{E }} + \, 0/{8} \times {\text{BD }} + \, 0/0{9} \times {\text{A}}^{2} \, + \, 0/{6} \times {\text{B}}^{2} \, + { 2}/{3} \times {\text{C}}^{2} \, + \, 0/{5} \times {\text{D}}^{2} \, + { 2}/{2} \times {\text{E}}^{2} $$

### The impact of parameters on the removal of TPHs from SWM

Through the use of response surface plots or contour plots generated by RSM, it is possible to illustrate the interaction between variables and to pinpoint the most advantageous values for the variables.

**Figure 2:**
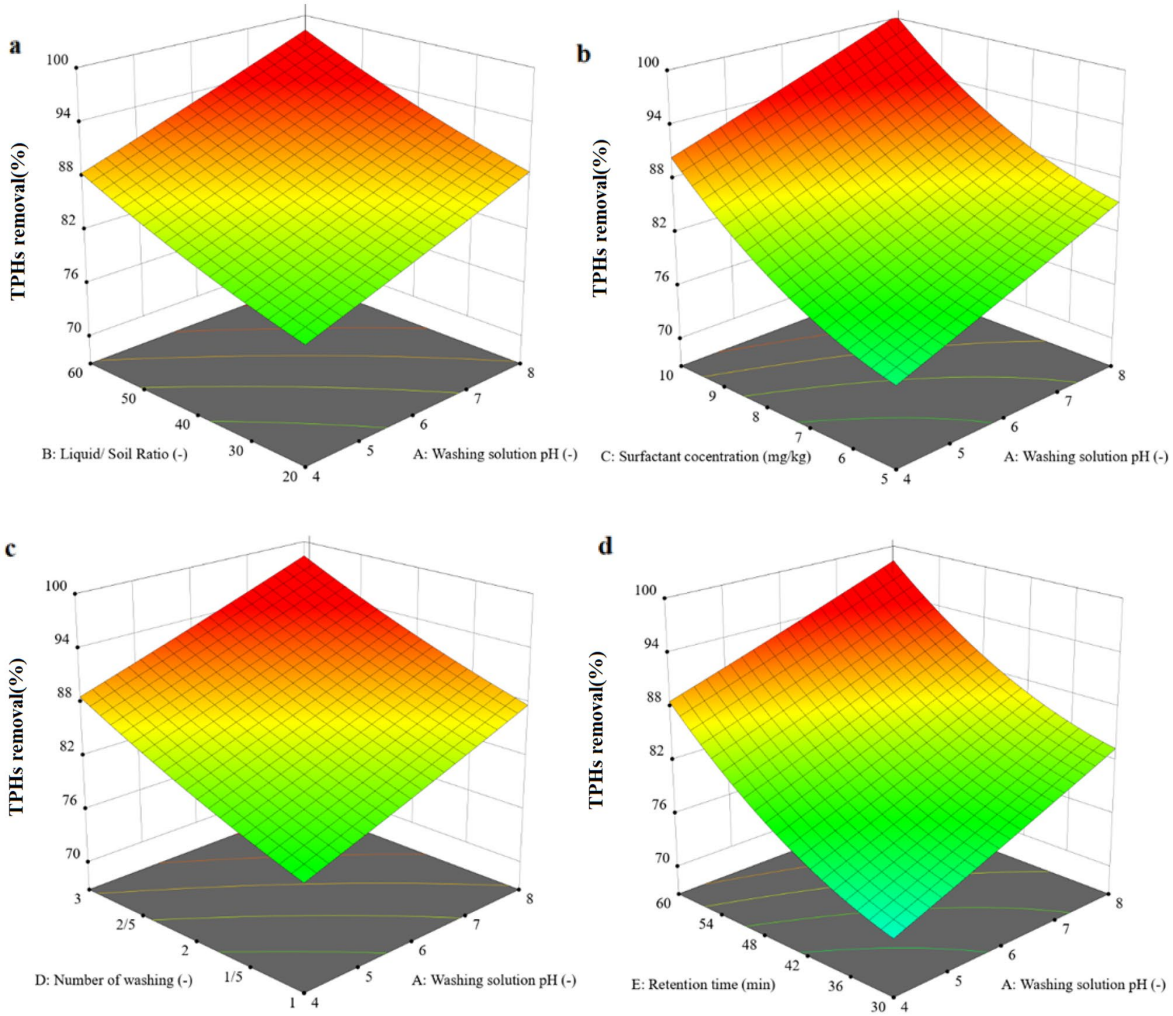
3D plots of significant interaction terms (washing solution pH, liquid/soil ratio, surfactant concentration, washing cycles, and retention time).

The 3D graph in (Fig. 2) displays the TPHs removal efficiency based on the five influence washing parameters in SWM. Each of the tested parameters, including washing solution pH, liquid/soil ratio, surfactant concentration, washing cycles, and retention time, had a substantial influence on TPHs removal efficiency.

#### Washing solution pH effect

The efficiency of SWM in TPHs removal is significantly influenced by the washing solution pH. The results of the effect of pH (4–8) on the efficiency of TPHs removal were surveyed and showed in (Fig. 2a). As the pH of the solution increased, the efficacy of the removal of TPHs increased significantly, with the highest efficacy at pH levels slightly basic and close to 8. The negative charges of organic and inorganic components of the soil colloid surfaces are increased by the increasing of the pH of the soil solution. This proliferation of negative charge will bring about the dispersion of soil particles and the desorption of organic pollutants from the colloid surface to the washing solution, owing to the repulsion between the negatively charged heads of organic matter and soil colloids. The solubility of organic matter increases with an increase in soil solution pH, since the main organic matter constituents (humic acid and fulvic acid) are soluble in alkaline solutions. When organic material is dissolved into the soil solution, the petroleum hydrocarbon molecules that are bound tightly are also released or made available for dissolution by surfactants by lowering surface tension^10^. According to a previous study conducted on diesel-contaminated soils, an increase in pH or alkalinity was reported, extracting more diesel than when in an acidic phase, and it was found that oils are more soluble under alkaline conditions than acidic^22^. In Liu and coworkers' study, remediation of diesel-contaminated soil at alkaline pH in the presence of tween 80 was better than other pH^23^. In addition, similar outcomes of the effect of pH were reported in Jiang and coworkers' study^24^.

#### Liquid/soil ratio effect

The effectiveness of SWM in the removal of TPHs was heavily affected by the liquid to soil ratio. The results of the effect of this parameter (20:1–60:1) on the efficiency of TPHs removal were surveyed and showed in (Fig. 2a). When the liquid to soil ratio enhanced, the elimination of TPHs significantly improved, with the highest effectiveness nearly accomplished at elevated levels. As anticipated, the application of a substantial amount of surfactant solution facilitates the TPHs removal. By having high liquid to soil ratio, greater TPHs concentrations are created between the two phases, thus intensifying the transfer of contaminants from the solid to the aqueous solution. Despite this, the cost of the procedure rises exponentially with the growth of the liquid to soil ratio, thus making it of utmost importance to identify the ideal value for this parameter^25^. Evaluation of the prior studies uncovered that the liquid to solid ratio was altered depending on the concentration and type of pollutants, the kind of surfactant, and the conditions of the soil washing process. In the study conducted by Lin and coworkers, the optimum ratio of liquid to soil was found to be 60:1^26^. Also, in Wang and coworkers study 60:1 ratio reported as a prepare ratio in the presence of EDTA^27^.

#### Surfactant concentration effect

The effectiveness of SWM in the removal of TPHs was heavily affected by the surfactant concentration. The results of the effect of this parameter (5–10 mg kg^−1^) on the efficiency of TPHs removal were surveyed and showed in (Fig. 2b). Surfactants are compounds that reduce the surface tension between two liquids or between a liquid and a solid, allowing for better mixing and dispersion of the solution. The concentration of surfactant used in soil washing can have a significant effect on the efficiency of the process. At low concentrations, surfactants may not be effective in removing contaminants from soil. This is because the surface tension of the water is not reduced enough to allow for effective mixing with the contaminants. As the concentration of surfactant increases, more contaminants are solubilized and removed from the soil^28,29^. However, at high concentrations, surfactants can become less effective due to factors such as foam formation or precipitation of the surfactant. Foam formation can occur when too much surfactant is added to the solution, leading to reduced contact between the solution and soil particles. Precipitation can occur when too much surfactant is added, causing it to bind with other compounds in the solution and become insoluble. Therefore, it is important to optimize the concentration of surfactant used in soil washing processes to ensure maximum efficiency while avoiding negative effects such as foam formation or precipitation. This optimization process may involve testing different concentrations of surfactant on contaminated soil samples and monitoring their effectiveness in removing contaminants^29^. Prior studies have found various levels of surfactants as the most favorable concentration, which is influenced by the kind and amount of pollutants in the soil, the type of surfactant and the retention time of the process^30,31^.

#### Washing cycles effect

The efficacy of SWM in the removal of TPHs was profoundly affected by the washing cycles. An examination of the impact of this parameter (1–3 time) on the effectiveness of TPHs removal was conducted and depicted in (Fig. 2c). Generally, the more times the soil washing is performed, the more successful it will be. Every washing cycle eliminates a certain amount of pollutants from the surface, and multiple washings can contribute to the removal of more pollutants. However, there are also some potential drawbacks to multiple washings. For example, excessive washing can lead to the loss of valuable nutrients and organic matter from the soil. Repeated washing may not be practical or cost-effective in all situations. Overall, the optimal washing cycles for soil remediation will depend on a variety of factors, including the type and concentration of contaminants present in the soil, as well as site-specific conditions, such as soil texture and moisture content. A thorough site assessment and careful planning are essential for achieving effective and sustainable results with soil washing^7,32^. In Rui and coworkers' study, 3 time washing was reported as an optimum multi-step washing to remediation of pollutants^33^. Also, in Piccolo and coworkers' study, the highest performance to heavy metals removal was got in 3 times washing^34^.

#### Retention time effect

The potency of SWM in the removal of TPHs was deeply affected by the retention time. A study of the influence of the 30–60 min parameter on TPHs removal efficiency was performed and presented in (Fig. 2d). Soil washing requires a certain length of contact between the soil and washing solution, which is known as the retention time. The effect of retention time on soil washing depends on various factors, such as the type of contaminants present in the soil, the type of washing solution used, and the soil properties. Increasing retention time can improve the efficiency of soil washing by allowing more time for the contaminants to dissolve or desorb from the soil particles and be removed by the washing solution. However, there is a limit to how much retention time can be increased before diminishing returns are observed. Different pollutants may have different optimal retention times for effective removal. Increasing retention time may also increase the amount of water required for soil washing, which can lead to higher costs and potential environmental effects, such as increased water usage and discharge. Overall, while increasing retention time can improve soil washing efficiency sometimes, it is important to consider other factors such as cost-effectiveness and environmental effects when determining optimal retention times for specific soil remediation projects^28,35,36^. In Bianco and coworkers'^37^ and Kang and coworkers^38^ studies, 60 min reported as an optimum retention time to PAHs removal from soil.

### Predicted optimum condition

Finally, we used Design-Expert software to estimate the optimal value of experiment variables for TPHs removal by SWM. pH of solution 7.8, liquid to solid ratio 50, reaction time 52 min, surfactant concentration 7.9 mg kg^−1^ and washing cycles 3 times were an optimum condition of SWM. In this situation, 98.8% of TPHs removed from soil. According to the prediction of the software, more than 99.9% of TPHs can be removed in the mentioned conditions. This difference can be due to measurement errors, device accuracy, the presence of interfering agents, etc. The reaction time had highest effect on SWM (7.7), then the surfactant concentration (7.4), the pH of solution (5.04), liquid to solid ratio (50.2) were effective respectively, and washing cycles (4.6) had lowest impact on SWM.

### Supplementary investigation

#### Reaction kinetics

The TPHs removal from the soil in the optimal condition of the SWM was showed by first- and second-order model used were expressed by the [Eqs. (4) and (5)]. The results of kinetics study at optimum condition were presented in (Table 4).

**Table 4:** First- and second-order coefficient.

Kinetics	K (min)	R^2^
First-order kinetic	0.0275	0.96
Second-order kinetic	0.0047	0.89

The first-order model gave a slightly better fit to the experimental results than the second-order model, based on the square regression coefficient R^2^. The removal of TPHs from the soil was regulated by the first-order kinetic model. Similarly, Li and coworkers^39^ study, and Khalladi and coworkers^14^ studies, first-order kinetic reported as a best model of fitting.

#### Effect of temperature, mixing speed, air flow and sonication on SWM performance

**Figure 3:**
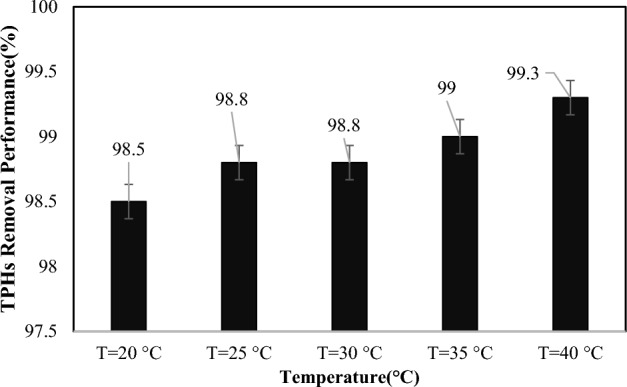
The effect of temperature on TPHs removal in SWM (pH 7.8, L/S ratio = 50:1, surfactant concentration = 7.9 mg L^−1^, washing cycles = 3 times, and retention time = 52 min).

The effect of reactor temperature on TPHs removal performance in SWM at 20, 25, 30, 35, and 40 ºC investigated. The results illustrated in (Fig. 3). Temperature can have a significant impact on the efficiency and effectiveness of soil washing processes. Higher temperatures can increase the rate of chemical reactions and solubility of contaminants in the soil, leading to faster and more thorough removal of pollutants^33^. However, excessively high temperatures can also cause problems such as increased vaporization of water and chemicals, which can lead to loss of materials and reduced efficiency. Additionally, high temperatures may also cause changes in the physical properties of the soil, such as increased viscosity or decreased permeability, which can hinder the washing process^40^. Therefore, it is important to control and optimize the temperature carefully during soil washing processes to ensure maximum effectiveness while minimizing any negative effects. The analysis of (Fig. 3) shows that, within the tested temperature range, the removal performance is not significantly affected, though a minor variation is present. The temperature dependent processes control the removal, desorption, and dissolution of elements, whereas the separation of incrustation or soil-trapped TPHs is mainly determined by mechanical conditions. Consequently, this test has revealed that other parameters in TPHs could have a greater impact than temperature. In Peng and coworkers' study, similar results were reported^25^.

**Figure 4:**
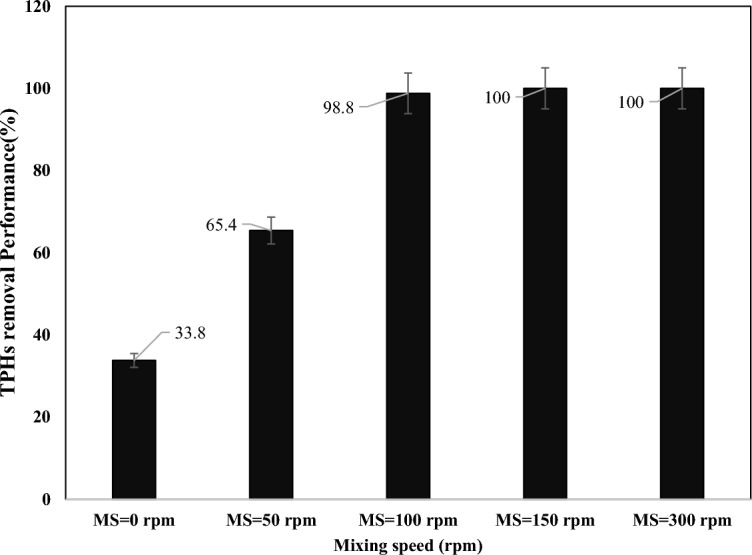
The effect of mixing speed on TPHs removal in SWM (pH 7.8, L/S ratio = 50:1, surfactant concentration = 7.9 mg L^−1^, washing cycles = 3 times, and retention time = 52 min).

The effect of mixing speed on TPHs removal performance in SWM at 0, 50, 100, 150, and 300 rpm investigated. The results illustrated in (Fig. 4). The process involves mixing the contaminated soil with water and other chemicals to extract the contaminants. Therefore, the mixing speed plays a crucial role in the soil washing process performance. The effect of mixing speed on soil washing process can be summarized as follows^35^:

1. Extraction efficiency: The extraction efficiency of contaminants from soil increases with increasing mixing speed. This is because higher mixing speeds create more turbulence, which enhances the contact between the soil particles and the washing solution, resulting in better extraction of contaminants.
2. Soil particle size: The effect of mixing speed on soil particle size depends on the type of soil being washed. Higher mixing speeds break down larger particles into smaller ones, which can increase the surface area available for contact with the washing solution.
3. Washing time: Higher mixing speeds can reduce the required washing time by increasing the rate at which contaminants are extracted from the soil.
4. Energy consumption: Higher mixing speeds require more energy, which can increase operating costs and environmental impacts.
5. Equipment design: The design of equipment used for soil washing must consider the effect of mixing speed on extraction efficiency, particle size, and energy consumption.

In conclusion, optimizing mixing speed is critical for achieving effective soil washing results. The appropriate mixing speed will depend on factors such as soil type, contaminant type and concentration, equipment design, and desired remediation goals. The findings of this study showed that the lowest performance was at zero mixing speed and the TPHs removal improved with the increasing of mixing speed until 150 rpm and then the efficiency stayed the same. In Ayele and coworkers study, similar results reported^41^.

**Figure 5:**
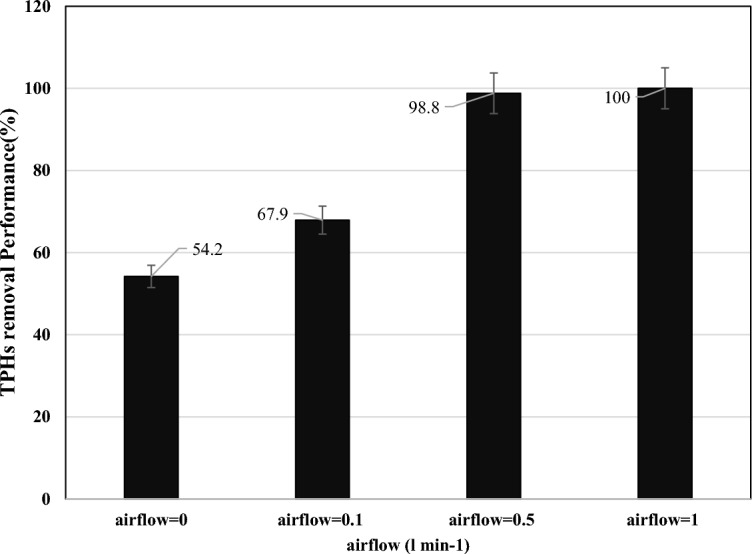
The effect of airflow rate on TPHs removal in SWM (pH 7.8, L/S ratio = 50:1, surfactant concentration = 7.9 mg L^−1^, washing cycles = 3 times, and retention time = 52 min).

The effect of airflow entering the reactor on TPHs removal performance in SWM at 0, 0.1, 0.5 and 1 L min^−1^ investigated (Fig. 5). Airflow can have both positive and negative effects on soil washing performance. On the one hand, airflow can help to increase the efficiency of soil washing by promoting the movement of water and contaminants through the soil matrix. This is because air can help to break up soil particles and create pathways for water to flow more easily. Too much airflow can also cause problems during soil washing. For example, if there is too much air entering the system, it can cause excessive turbulence in the water and lead to increased erosion of the soil matrix. This can cause a loss of valuable topsoil and a reduced overall effectiveness of the washing process. Overall, it is important to carefully control airflow during soil washing operations in order to optimize performance and minimize negative effects on soil quality^11^. In this current study, results indicated that by increasing the amount of aeration, the efficiency of the process increases.

When the airflow rate was 0, 0.1, 0.5, and 1 L min^−1^, the efficiency of the process was 54.2, 67.9, 99.8, and 100%, respectively.

The results show that the increase of airflow rates up to 0.5 L min^−1^ causes a significant increase in efficiency, but the efficiency is close to the values of 0.5 and 1 L min^−1^, and considering the energy consumed by the aeration pump, the value of 0.5 L min^−1^ was chosen as the optimal inlet air flow rate. In Tran and coworkers' study, airflow rate was investigated as an one of the major important parameters^11^. In Ayele and coworkers study reported that increase in air flow rate will result in more hydrophobic microbubbles being generated. So, the airflow has an impact on soil washing performance^41^.

**Figure 6:**
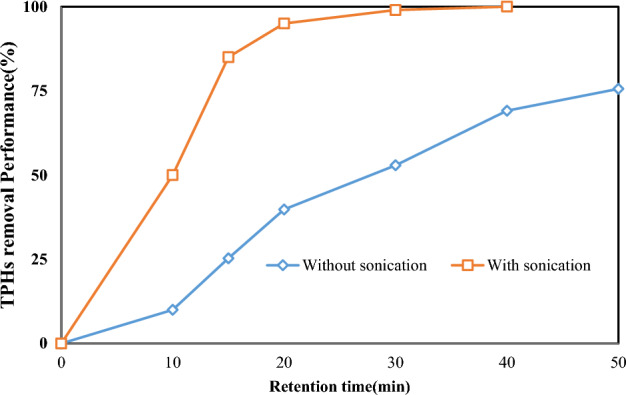
The effect of airflow rate on TPHs removal in SWM (pH 7.8, L/S ratio = 50:1, surfactant concentration = 7.9 mg L^−1^, washing cycles = 3 times, and retention time = 52 min).

A study was done to assess the effect of the sonication mechanism on TPHs removal performance in SWM (Fig. 6). Sonication technology is an environmentally friendly and clean approach to treating and degrading toxic organic pollutants. Sonication is a process that uses high-frequency sound waves to agitate and dislodge particles from surfaces^42^. In soil washing, sonication can enhance the removal of contaminants from soil particles. The effect of the sonication mechanism on soil washing process can think by frequency, amplitude, duration, and soil type.

Using sonication enhances the rate of leaching and improves the removal efficiency by diffusion into the outermost layer. The utilization of ultrasonic processes for leaching produces higher removal efficiency with a quicker processing time. Sonication technology applied to soil systems might enhance the desorption of pollutants by disintegrating the soil structure. The conceptual dependence of desorption of contaminants from the soil surface is largely determined by changes in Gibbs energy (∆G°) of a system^43^. In this study, in the presence of ultrasonic waves, the removal rate of TPHs occurs in a shorter time. Also, in a one-step wash, all of TPHs were separated from the soil. In Effendi and coworkers study, the effect of ultrasonic mechanism to remediation of polluted soil was investigated^44^.

#### Effect of soil washing on physico-chemical soil properties

**Figure 7:**
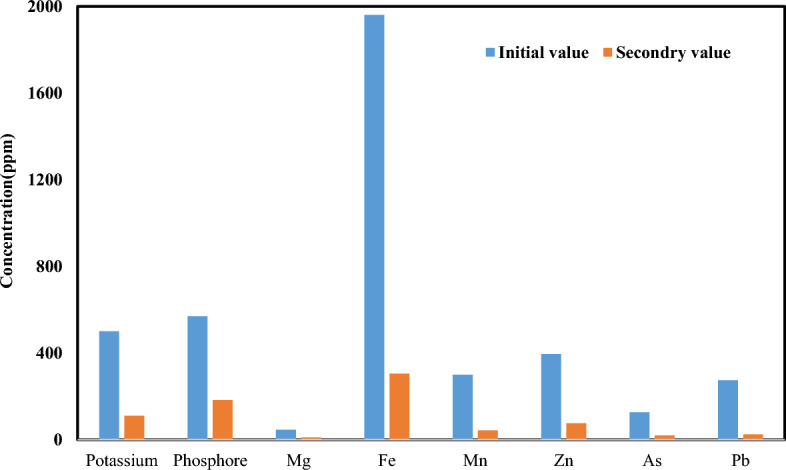
Changes of trace and major elements during soil washing.

We evaluated the effect of SWM on the physical and chemical characteristics of the soil in an optimal condition (Fig. 7). The effect of soil washing on physico-chemical soil properties depends on several factors, including the type and concentration of contaminants, the type of washing solution used, and the duration and intensity of washing. SWM can alter soil texture by removing fine particles such as clay and silt, which are more likely to be contaminated than coarser sand particles. This can cause changes in soil structure, porosity, and water-holding capacity. SWM can change soil pH by removing or adding ions that affect acidity or alkalinity. SWM can remove organic matter and nutrients along with contaminants if the washing solution is too strong or if the duration of washing is too long and can reduce cation exchange capacity (CEC) by removing cation-exchange sites along with contaminants^45^. Based on results, significant changes were observed in the measured values. The soil pH was increased from 7.21 to 8.29 and CEC was also reduced from 2.47 to 1.95 (cmol/kg). In Bari and coworkers' study, similar results were reported^46^.

In the end, in Table 5, different processes are presented in remediation soil contaminated with petroleum hydrocarbons.

**Table 5:** Various studies in petroleum hydrocarbons removal from soil.

Process	Methods	Results	Author and references
FeS@BC activated persulfate	The ratio of FeS to biochar mass, the amount of PS, the amount of FeS@BC, and the initial pH were studied	There was a 61.83% TPH removal rate for the FeS@BC/PS system, which was significantly higher than the FeS/PS system's 47.91% removal rate	Xia^47^
Microbial removal	Use acinetobacter baumannii strain JYZ-03	Eliminated 93.29% of the diesel oil amount over a period of 7 days	Su^48^
Bioremediation	Use *Bacillus amyloliquefaciens* A3	removed 45.44% of petroleum hydrocarbons	Wang^49^
Fe/N co-doped biochar-mediated heterogeneous Fenton	Catalyst was fabricated by sequential pyrolysis, and hydrothermal methods and critical parameters investigated	The employed system demonstrated the highest effectiveness for total petroleum hydrocarbons (TPHs) removal with 91.4% efficiency	Li^50^
Soil washing method	Main parameters optimized via CCD	pH of 7.8, liquid to solid ratio of 50:1, reaction time of 52 min, surfactant concentration of 7.9 mg kg^−1^ and washing cycles of three times was optimum condition that 98.8% of TPHs were removed from the soil	Current study

## Conclusion

This study optimized the performance of SWM to remove TPHs from a real contaminated soil sample. After sampling and determining the physico-chemical characteristics of the soil, optimization of variables affecting the efficiency of the process was done by the BBD method. Based on the BBD design and the correlation coefficients and other indicators, the quadratic model was proposed. The minimum, maximum and mean removal of TPHs during the stages of the study were 63.5, 94.5 and 76.7%, respectively. Based on ANOVA and indices F-value (1064.5) and p-value (0.0001), there was a high correlation between the variables and the proposed model was significant. The best results of SWM were achieved by pH of 7.8, liquid to solid ratio of 50:1, reaction time of 52 min, surfactant concentration of 7.9 mg kg^−1^ and washing cycles of three times. 98.8% of TPHs were removed from the soil under this circumstance. Based on the kinetic results, the kinetic of TPHs removal follows the first-order kinetics (R^2^ = 0.96). The efficiency of the process was not affected significantly by temperature. The removal efficiency intensified from 0 to 150 rpm and then sustained its steady state. The incoming air flow improved the removal efficiency, and ultrasonic waves in the reaction environment improved the process efficiency and reduced the process retention time and the washing cycles times. After the physico-chemical analysis of the washed soil, it was found that the concentration of other elements has decreased significantly.

### Supplementary Information

Supplementary Tables.

## Data Availability

The datasets generated and analyzed during the current study available from the corresponding author on reasonable request.
