# Supplementary material for: Optimisation of soil washing method for removal of petroleum hydrocarbons from contaminated soil around oil storage tanks using response surface methodology
Source: Sci Rep. 2023 Sep 19;13:15457. doi: 10.1038/s41598-023-42777-9 (PMC10509228; doi:10.1038/s41598-023-42777-9)
Supplement: Supplementary file 1 — Supplementary Tables. [file 41598_2023_42777_MOESM1_ESM.docx]

**Table. 1S: Specifications of the sampling location**

| **Code** | **Geographic coordinates** | **Above sea level (m)** |
| --- | --- | --- |
| S1 | 35º32'19"N  51º25'46"E | 1021 |
| S2 | 35º32'19"N  51º25'50"E | 1022 |
| S3 | 35º32'19"N  51º25'54"E | 1023 |

**Table. 2 S: PHs removal from soil via SWM based on BBD design**

|  | Factor 1 | Factor 2 | Factor 3 | Factor 4 | Factor 5 | Response 1 |
| --- | --- | --- | --- | --- | --- | --- |
| Run | A:Washing solution pH | B:Liquid/ Soil Ratio | C:Surfactant cocentration | D:Number of washing | E:Retention time | PHs |
|  | - | - | mg/kg | - | min |  |
| 1 | 4 | 20 | 7/5 | 2 | 45 | 65/5 |
| 2 | 6 | 60 | 5 | 2 | 45 | 75 |
| 3 | 6 | 40 | 10 | 3 | 45 | 90 |
| 4 | 8 | 40 | 7/5 | 3 | 45 | 85 |
| 5 | 6 | 20 | 7/5 | 1 | 45 | 66/9 |
| 6 | 6 | 20 | 7/5 | 2 | 60 | 80 |
| 7 | 6 | 60 | 7/5 | 1 | 45 | 76/6 |
| 8 | 6 | 40 | 10 | 2 | 30 | 78/6 |
| 9 | 8 | 40 | 7/5 | 2 | 30 | 75 |
| 10 | 4 | 60 | 7/5 | 2 | 45 | 75 |
| 11 | 6 | 40 | 10 | 1 | 45 | 80 |
| 12 | 6 | 40 | 5 | 2 | 60 | 80 |
| 13 | 6 | 40 | 7/5 | 2 | 45 | 75 |
| 14 | 4 | 40 | 5 | 2 | 45 | 65 |
| 15 | 6 | 20 | 7/5 | 2 | 30 | 65 |
| 16 | 4 | 40 | 7/5 | 3 | 45 | 75 |
| 17 | 6 | 60 | 7/5 | 3 | 45 | 86/9 |
| 18 | 8 | 40 | 5 | 2 | 45 | 75 |
| 19 | 6 | 40 | 7/5 | 2 | 45 | 75 |
| 20 | 6 | 40 | 5 | 3 | 45 | 75 |
| 21 | 4 | 40 | 10 | 2 | 45 | 80 |
| 22 | 6 | 40 | 7/5 | 2 | 45 | 75 |
| 23 | 6 | 40 | 10 | 2 | 60 | 95 |
| 24 | 6 | 40 | 7/5 | 3 | 30 | 75 |
| 25 | 8 | 60 | 7/5 | 2 | 45 | 85 |
| 26 | 6 | 20 | 5 | 2 | 45 | 65 |
| 27 | 6 | 40 | 7/5 | 1 | 60 | 80 |
| 28 | 6 | 20 | 10 | 2 | 45 | 80 |
| 29 | 4 | 40 | 7/5 | 2 | 30 | 64/2 |
| 30 | 6 | 40 | 5 | 1 | 45 | 65 |
| 31 | 6 | 40 | 7/5 | 2 | 45 | 75 |
| 32 | 6 | 60 | 7/5 | 2 | 60 | 90 |
| 33 | 6 | 20 | 7/5 | 3 | 45 | 75 |
| 34 | 4 | 40 | 7/5 | 1 | 45 | 65 |
| 35 | 6 | 40 | 7/5 | 2 | 45 | 75 |
| 36 | 6 | 60 | 10 | 2 | 45 | 90 |
| 37 | 8 | 40 | 7/5 | 2 | 60 | 90 |
| 38 | 4 | 40 | 7/5 | 2 | 60 | 80 |
| 39 | 8 | 40 | 7/5 | 1 | 45 | 75 |
| 40 | 8 | 20 | 7/5 | 2 | 45 | 75 |
| 41 | 6 | 40 | 7/5 | 3 | 60 | 90 |
| 42 | 6 | 40 | 5 | 2 | 30 | 63/5 |
| 43 | 6 | 40 | 7/5 | 2 | 45 | 75 |
| 44 | 8 | 40 | 10 | 2 | 45 | 90 |
| 45 | 6 | 40 | 7/5 | 1 | 30 | 65 |
| 46 | 6 | 60 | 7/5 | 2 | 30 | 75 |

**Table. 3 S: Analysis of variance**

| **Source** | **Sum of Squares** | **df** | **Mean Square** | **F-value** | **p-value** |  |
| --- | --- | --- | --- | --- | --- | --- |
| **Model** | 3132/62 | 20 | 156/63 | 1064/55 | < 0.0001 | significant |
| A-Washing solution pH | 407/03 | 1 | 407/03 | 2766/41 | < 0.0001 |  |
| B-Liquid/ Soil Ratio | 404/01 | 1 | 404/01 | 2745/88 | < 0.0001 |  |
| C-Surfactant concentration | 885/06 | 1 | 885/06 | 6015/38 | < 0.0001 |  |
| D-Number of washing | 395/02 | 1 | 395/02 | 2684/75 | < 0.0001 |  |
| E-Retention time | 957/90 | 1 | 957/90 | 6510/44 | < 0.0001 |  |
| AB | 0/0625 | 1 | 0/0625 | 0/4248 | 0/5205 |  |
| AC | 0/0400 | 1 | 0/0400 | 0/2719 | 0/6067 |  |
| AD | 0/0100 | 1 | 0/0100 | 0/0680 | 0/7965 |  |
| AE | 0/3600 | 1 | 0/3600 | 2/45 | 0/1303 |  |
| BC | 0/0225 | 1 | 0/0225 | 0/1529 | 0/6991 |  |
| BD | 2/89 | 1 | 2/89 | 19/64 | 0/0002 |  |
| BE | 0/0100 | 1 | 0/0100 | 0/0680 | 0/7965 |  |
| CD | 0/0625 | 1 | 0/0625 | 0/4248 | 0/5205 |  |
| CE | 0/0900 | 1 | 0/0900 | 0/6117 | 0/4415 |  |
| DE | 0/0100 | 1 | 0/0100 | 0/0680 | 0/7965 |  |
| A² | 0/0837 | 1 | 0/0837 | 0/5687 | 0/4578 |  |
| B² | 3/21 | 1 | 3/21 | 21/80 | < 0.0001 |  |
| C² | 46/42 | 1 | 46/42 | 315/49 | < 0.0001 |  |
| D² | 2/78 | 1 | 2/78 | 18/91 | 0/0002 |  |
| E² | 44/43 | 1 | 44/43 | 301/95 | < 0.0001 |  |
| **Residual** | 3/68 | 25 | 0/1471 |  |  |  |
| Lack of Fit | 1/80 | 20 | 0/0902 | 0/2404 | 0/9906 | not significant |
| Pure Error | 1/88 | 5 | 0/3750 |  |  |  |
| **Cor Total** | 3136/30 | 45 |  |  |  |  |
